# Comparative Effects of Cigarette Smoke and E-Cigarette Vapor on Oral Squamous Cell Carcinoma: Unraveling Distinct Molecular Pathways

**DOI:** 10.26502/droh.0098

**Published:** 2025-09-08

**Authors:** Ryan Kinney, Logan Ponder, Neha Patel, Ankita Chatterjee, Kristina Vu, Harishma Sidhu, Benjamin Bikman, Paul R Reynolds, Juan A Arroyo

**Affiliations:** 1 College of Dental Medicine, Roseman University of Health Sciences, South Jordan, UT, USA; 2 Department of Cell Biology and Physiology, Brigham Young University, Provo, UT, USA

**Keywords:** Oral squamous cell carcinoma, Cigarette smoke extract, e-cigarette vapor extract, RAGE, NF-κB

## Abstract

Oral squamous cell carcinoma (OSCC) poses a significant global health burden, driven by cigarette smoking and complicated by the rising popularity of e-cigarettes. This study compares the effects of cigarette smoke extract (CSE) and e-cigarette vapor extract (EVE) on OSCC progression using Ca9-22 gingival OSCC cells. CSE significantly increased cell invasion by upregulating the receptor for advanced glycation end-products (RAGE), nuclear factor kappa B (NF-κB), and matrix metalloproteinases (MMP-9, MMP-13), facilitating extracellular matrix degradation and metastasis. Conversely, the effects of EVE varied by flavor and nicotine content. Red Hot EVE (cinnamaldehyde-based) enhanced invasion and NF-κB levels with nicotine while reducing MMP-9 expression, suggesting alternative invasion pathways via MMP-13. Green Apple EVE had a less pronounced impact, indicating flavor-specific bioactivity. These findings reveal distinct mechanisms: CSE drives RAGE-NF-κB-MMP-mediated invasion, whereas EVE elicits variable, flavor-dependent responses, potentially involving nicotinic acetylcholine receptors. Targeted therapies, such as RAGE or NF-κB inhibitors, could mitigate tobacco-related OSCC progression. With e-cigarette use surging among youth, stricter regulations on flavored products and robust public health campaigns are urgently needed. Future research should investigate chronic exposures and diverse OSCC models to refine clinical and regulatory approaches, addressing the evolving landscape of tobacco-related oral cancers.

## Introduction

Oral squamous cell carcinoma (OSCC) is a primary global health concern. It is recognized as the sixth most common malignancy worldwide, with an estimated incidence of over 300,000 new cases annually and a 5-year survival rate ranging from 20–60% [1,2]. This poor prognosis is primarily attributed to the high rates of recurrence (up to 50%) and early metastatic spread to regional lymph nodes and distant organs, which complicates treatment and reduces survival outcomes [3,4]. OSCC predominantly arises in the oral cavity, including the tongue, gingiva, and floor of the mouth, and is strongly associated with environmental and lifestyle risk factors. Among these, tobacco use, particularly cigarette smoking, is a well-documented etiological factor, increasing the risk of OSCC development by a factor of 7–10 times compared to non-smokers [5,6]. Cigarette smoke contains a complex mixture of over 7,000 chemicals, including potent carcinogens such as polycyclic aromatic hydrocarbons, tobacco-specific nitrosamines, and reactive oxygen species [7]. These compounds induce chronic inflammation, DNA damage, and cellular invasion, critical to oncogenic transformation and tumor progression [8,9]. The receptor for advanced glycation end-products (RAGE), a pattern-recognition receptor, is pivotal in OSCC pathogenesis under cigarette smoke exposure. RAGE activation triggers downstream signaling cascades involving Ras, AKT, and nuclear factor kappa B (NF-κB), which promote the expression of matrix metalloproteinases (MMPs) such as MMP-2, MMP-9, and MMP-14 [10,11]. These enzymes degrade extracellular matrix components, facilitating cancer cell invasion and metastasis, thereby exacerbating disease severity [12].

The advent of electronic cigarettes (e-cigarettes) has introduced a novel dimension to tobacco-related health risks, with their use rapidly gaining popularity, particularly among adolescents and young adults [13]. Marketed as a safer alternative to traditional cigarettes, e-cigarettes deliver aerosolized nicotine, flavorings, and other chemicals through a heating mechanism, avoiding the combustion-associated toxins found in cigarette smoke [14]. However, the long-term health implications of e-cigarette use, particularly on oral health and OSCC risk, remain poorly understood due to limited longitudinal data [15]. E-cigarette vapor contains volatile organic compounds, heavy metals (e.g., nickel, lead), and flavoring agents such as cinnamaldehyde, diacetyl, and menthol, which have been shown to induce oxidative stress, inflammation, and cytotoxicity in various cell types [16,17]. For instance, cinnamaldehyde, a common e-cigarette flavoring, has been linked to pro-inflammatory responses, while diacetyl is associated with epithelial cell damage [18]. Emerging evidence indicates that e-cigarette vapor extract (EVE) modulates cancer cell behavior through pathways distinct from those activated by cigarette smoke extract (CSE). Specifically, EVE exposure has been shown to influence inflammatory markers such as tumor necrosis factor-alpha (TNF-α), c-Jun N-terminal kinase (JNK), and NF-κB in OSCC cells, with effects varying by flavor and cell type [19]. These findings raise concerns about the potential oncogenic risks of e-cigarettes, particularly as their use continues to rise globally, with prevalence rates reaching up to 8.2% among adults and significantly higher among adolescents [20,21].

The molecular mechanisms underlying the effects of CSE and EVE on OSCC are complex and appear to be influenced by their distinct chemical compositions. CSE primarily drives RAGE-dependent invasion by activating Ras, AKT, and NF-κB, increasing MMP expression, and enhancing cellular invasiveness [22]. In contrast, EVEappears to elicit flavor- and cell type-specific responses, with certain flavors (e.g., cinnamaldehyde-based Red Hot) promoting TNF-α and MMP-13 expression in gingival OSCC cells (Ca9-22), while others (e.g., apple-based flavors) may preferentially activate NF-κB and JNK pathways [22]. These differential effects of flavoring agents suggest that the chemical constituents of e-cigarette vapor, including nicotine and flavoring agents, may interact with OSCC cells in a manner distinct from traditional cigarette smoke, potentially engaging alternative signaling pathways such as mitogen-activated protein kinases (MAPKs) or stress-related cascades [23]. Furthermore, the role of nicotine, a common component in both CSE and EVE, appears to amplify these effects, enhancing inflammatory and invasive responses in a context-dependent manner [24].

Given the increasing prevalence of e-cigarette use and the persistent burden of cigarette smoking, understanding the comparative impacts of CSE and EVE on OSCC progression is of critical importance. This is particularly relevant in the context of younger demographics and in populations with high exposure to second-hand smoke, which also upregulates RAGE and promotes OSCC invasion [25,26]. This study hypothesizes that CSE and EVE differentially regulate OSCC cell invasion and inflammation due to their unique chemical profiles. By synthesizing data from studies on CSE and EVE, this manuscript aims to elucidate the molecular and cellular mechanisms underlying their effects on OSCC, focusing on identifying potential therapeutic targets, such as RAGE inhibitors or anti-inflammatory agents, to mitigate tobacco-related oral carcinogenesis.

## 2.0 Materials and Methods

### 2.1 Cell Culture and Treatments

Ca9-22 human gingival OSCC cells (ATCC, Manassas, VA, USA) were used for these experiments. Cells were cultured in Roswell Park Memorial Institute (RPMI) medium supplemented with 10% fetal bovine serum (FBS; Invitrogen) at 37°C in a 5% CO_2_ atmosphere. Cells were treated with cigarette smoke extract (CSE). CSE was produced by continuously smoking a single 2RF4 research cigarette (2RF4 cigarette contains 11.7 mg of total particulate matter, 9.7 mg of tar, and 0.85 mg of nicotine; University of Kentucky, Lexington, KY, USA). The cigarette’s filtered end was attached to a vacuum pump, which drew the smoke particles into 5 ml of RPMI medium. This solution was designated 100% cigarette smoke extract and diluted to 0.05% for experiments. Ca9-22 cells were treated with 0.05% CSE or control media for 24 hours. The 0.05% CSE was chosen from preliminary studies, which showed this concentration as the lowest needed to exert a response. EVE was generated by vaporizing Red Hot (8Ohm1, Inc.) or Green Apple (Daze Mfg.) e-cigarette liquids with or without 6 mg nicotine. To make EVE, the eCig module was attached to a vacuum pump via its mouthpiece, with the button on the module held down for 3 seconds. The pump extracted vapor through a pipette tip immersed in a tube with 10 ml of serum-free medium. This cycle, consisting of 3-second puffs followed by 20-second pauses, was repeated for 20 puffs. The resulting medium was designated as 100% EVE solution.

This was diluted to 10% for treatments, and cells were exposed for 6 hours (EVE concentration and treatment time were chosen from preliminary studies showing these to be the lowest needed to exert a response.

### 2.2 Real-Time Cell Invasion Assay

Real-time cell invasion of OSCC cells was measured as previously done in our laboratory [8]. Briefly, following experimental treatments, invasion was determined using an xCELLigence RTCA DP instrument (ACEA Biosciences). Invasion was assessed in 16-well CIM-Plates (ACEA Biosciences) (n = 10), where cells were plated in the top chamber at a concentration of 20,000 cells per well in RPMI containing 2% FBS. The bottom chamber contained RPMI supplemented with 10% FBS. The cells were then placed in the xCelligence RTCA DP instrument, and invasion readings were obtained every 15 min for 24 h.

### 2.3 Immunoblotting

Western blot analysis was performed to quantify protein expression. Cells were lysed (n=10) in Radioimmunoprecipitation Assay (RIPA) buffer (Fisher Scientific), and protein lysates (30 μg) were separated on Mini-PROTEAN TGX Precast gels (Bio-Rad Laboratories) and transferred to nitrocellulose membranes. Primary antibodies targeted RAGE, MMP-2, MMP-9, and β-actin (Cell Signaling). Membranes were incubated with fluorescent secondary antibodies and imaged using a Li-COR Odyssey CLx.

### 2.4 NF-κB levels

NF-κB levels in conditioned media were measured using colorimetric ELISA assays (n=4; Active Motif).

### 2.5 Statistical Analysis

Data were expressed as means ± standard error (SE). Protein expression and invasion indices differences were assessed by one- and two-way analysis of variance (ANOVA). Results with p-values <0.05 were considered significant. Statistical analyses were performed using GraphPad Prism 8.0 software.

## 3.0 Results

### 3.1 RAGE Protein Expression

**Figure 1:**
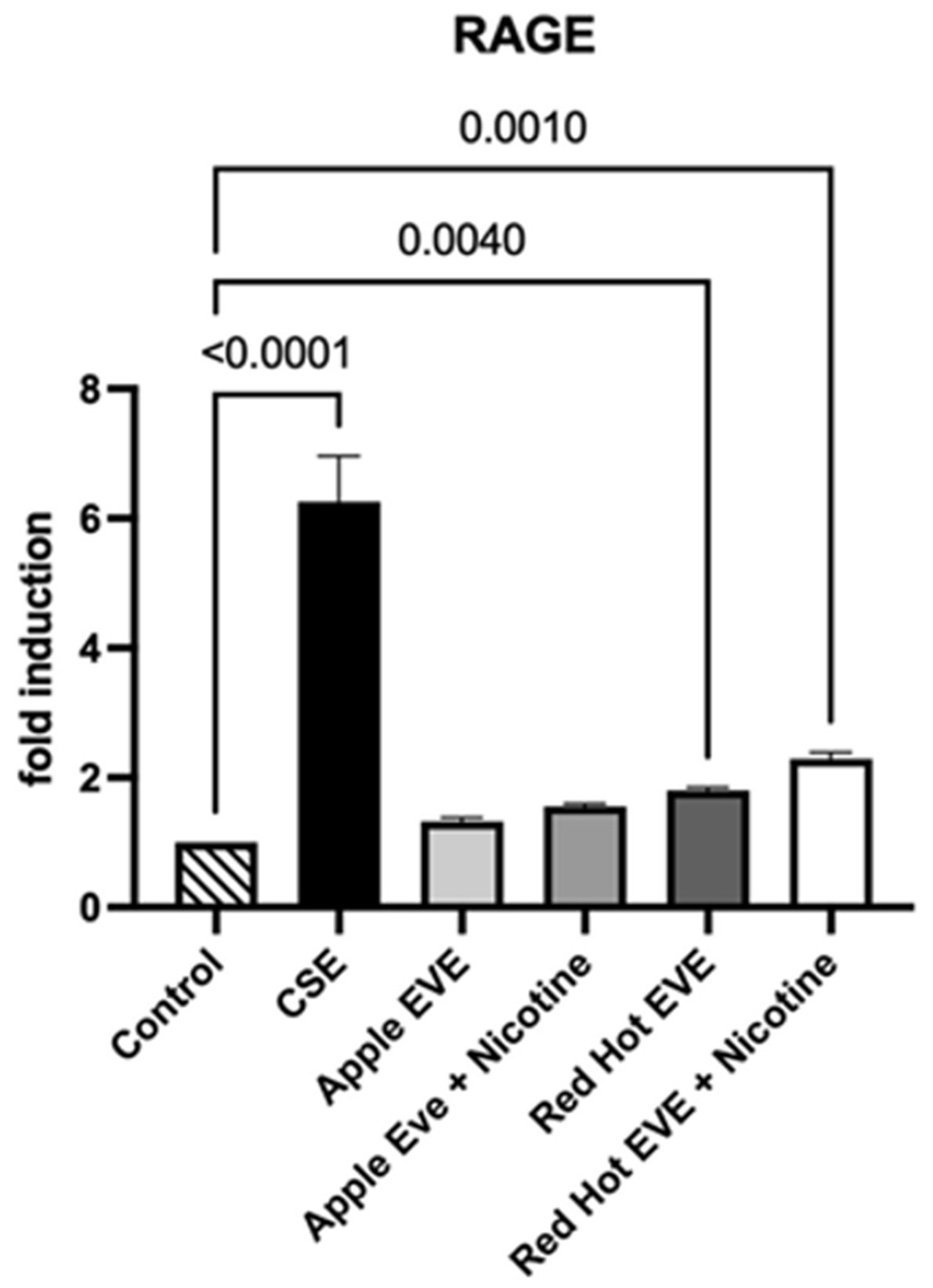
RAGE protein expression in Ca9-22 cells treated with CSE or EVE. (A) Representative Western blot showing RAGE and β-actin levels. (B) Quantifying RAGE expression, with significant increases in CSE and Red Hot EVE with nicotine treatments compared to controls (p ≤ 0.05).

We first evaluated RAGE receptor expression in Ca9-22 cells. RAGE signaling is involved in inflammatory, metabolic, and immune signaling [27,28]. 0.05% cigarette smoke extract (CSE) increased Ca9-22 cells’ invasion by 2.4-fold (p<0.001) as compared to controls. In contrast, Green Apple EVE-treated cells, with or without nicotine, did not significantly alter RAGE expression (Figure 1). Only Red Hot EVE and nicotine treatment affected Ca9-22 by increasing cell invasion (2.1-fold; p<0.001) when compared to controls (Figure 1).

### 3.2 NF-κB Levels

**Figure 2:**
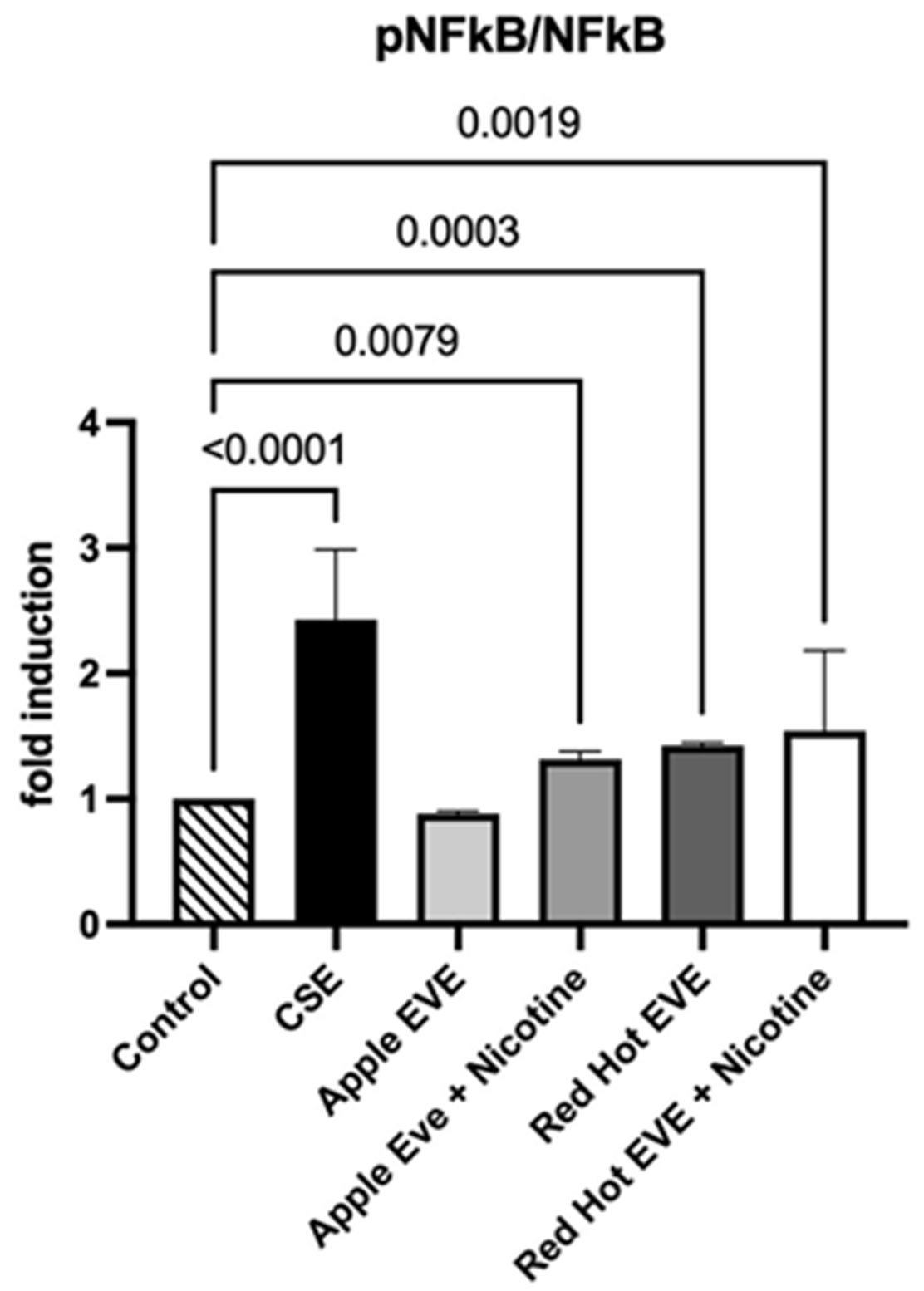
NF-κB levels in Ca9-22 cells treated with CSE or EVE. Colorimetric ELISA assays show elevated NF-κB in CSE, Red Hot EVE with or without nicotine, and Green Apple EVE without nicotine compared to controls (p ≤ 0.05).

RAGE activates NF-κB, a key transcription factor that drives inflammation by upregulating pro-inflammatory cytokines and matrix metalloproteinases in oral squamous cell carcinoma cells exposed to cigarette smoke. (Chapman et al., 2018) NF-κB levels in conditioned media from Ca9-22 cells were measured using colorimetric ELISA assays. Treatment with 0.05% CSE increased NF-κB levels by 2.4-fold compared to controls (P<0.01; Figure 2). Red Hot EVE with and without nicotine elevated NF-κB by ~1.4-fold (P<0.002), while Green Apple EVE without nicotine showed a 1.3-fold increase in NF-κB as compared to controls (P>0.008) (Figure 2).

### 3.3 Cell Invasion

**Figure 3:**
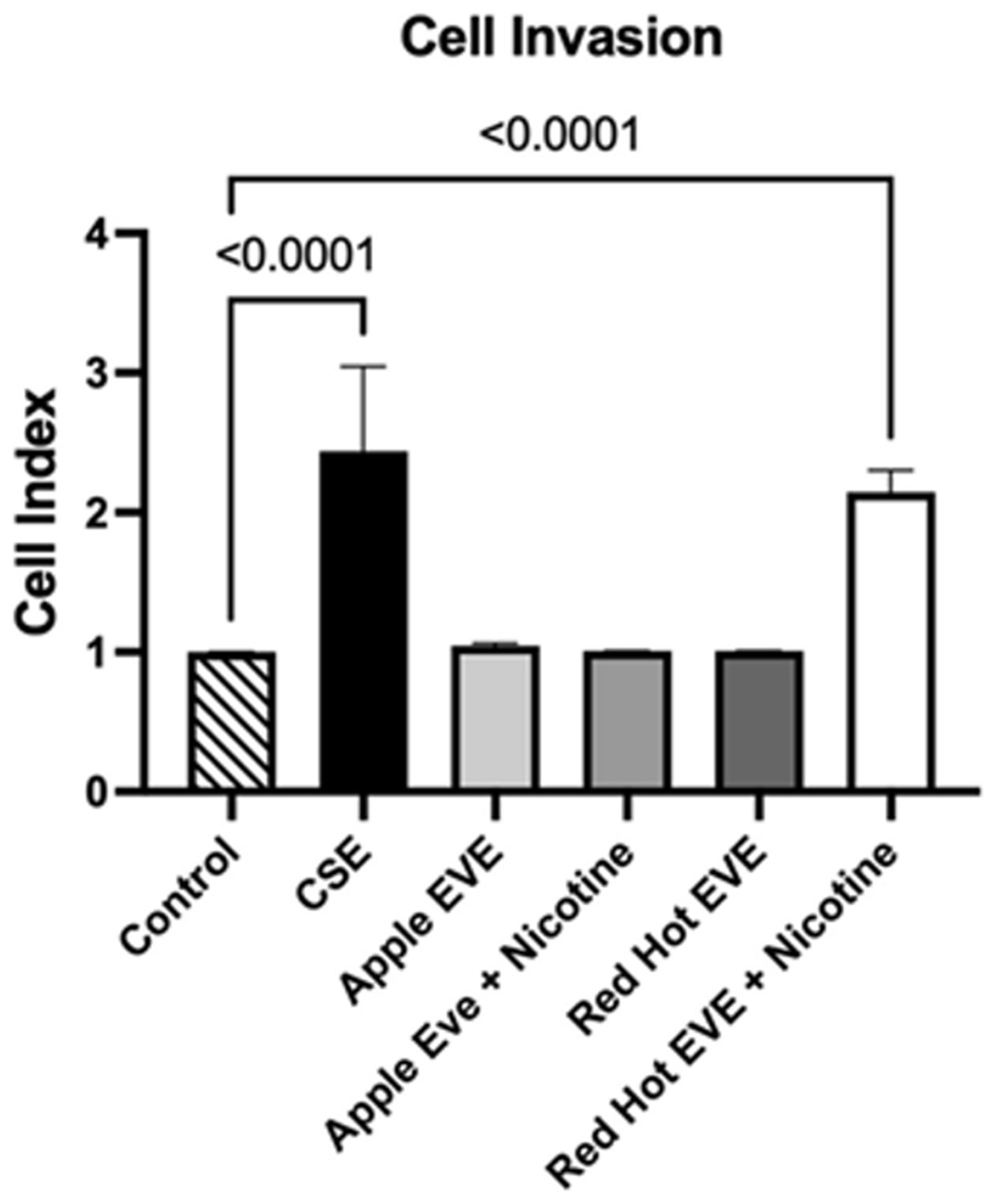
Ca9-22 invasion with CSE or EVE Treatments. CSE and Red Hot EVE + nicotine increased Ca9-22 invasion compared to controls. Significant differences are shown with p ≤ 0.05.

OSCC cell invasion is critical for tumor progression and metastasis, as it enables cancer cells to breach the extracellular matrix and spread to regional lymph nodes and distant organs, significantly worsening prognosis and contributing to high recurrence rates [3]. Real-time cell invasion was quantified using the xCELLigence RTCA DP instrument in Ca9-22 cells treated with 0.05% CSE or 10% EVE (Red Hot or Green Apple, with or without 6 mg nicotine). 0.05% CSE induced a 2.4-fold increase (P<0.0001) as compared to controls (Figure 3). Red Hot EVE resulted in a 2.1-fold increase relative to controls (P<0.0001; Figure 3). Green Apple EVE, with or without nicotine, did not significantly affect invasion (Figure 3).

### 3.4 MMP-9 Protein Expression

**Figure 4:**
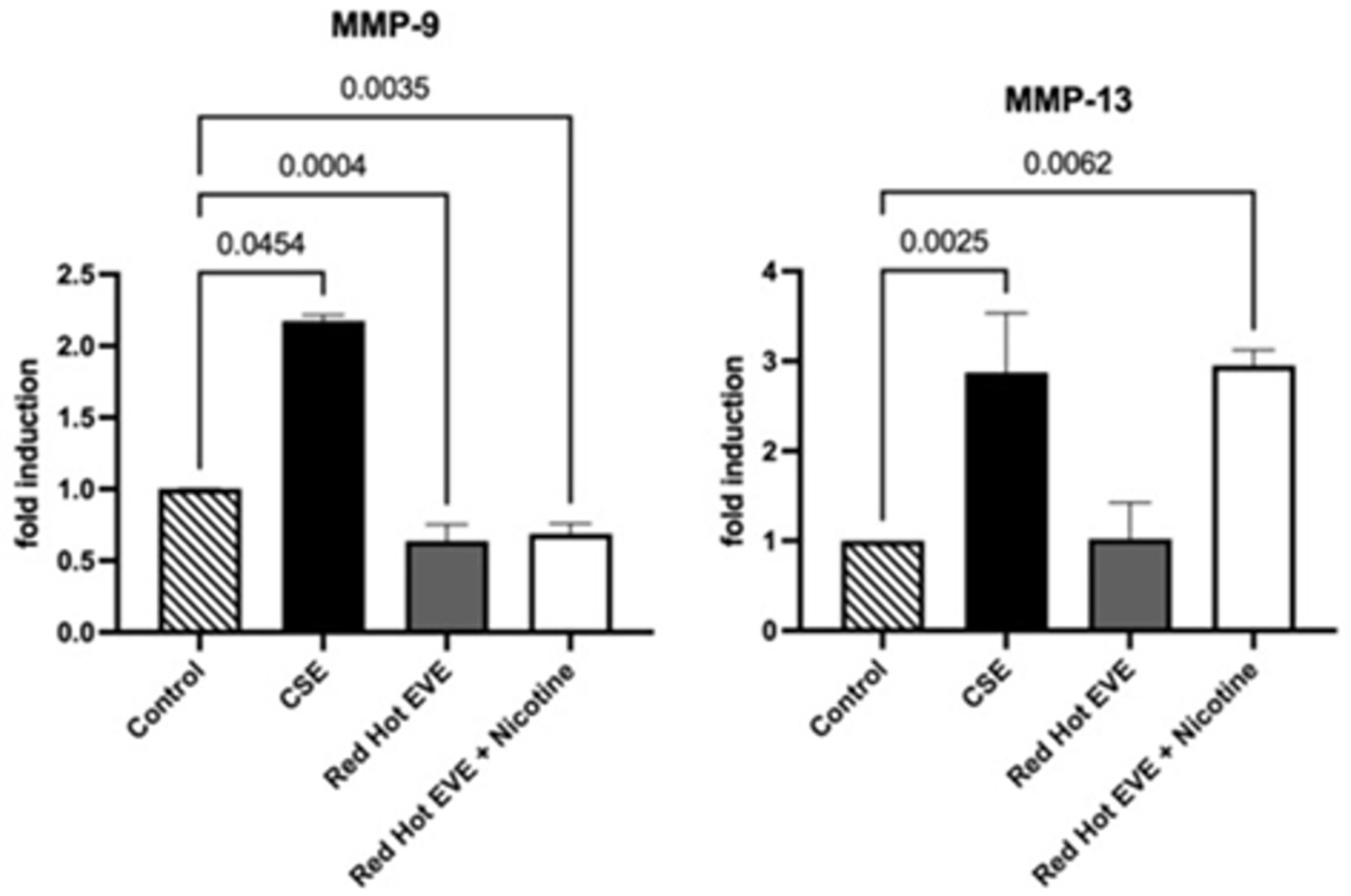
MMP-9 and MMP-13 during CSE or EVE treatment of Ca9-22 cells. A representative Ca9-22 MMP-9 and −13 western blot picture is shown in (A). Western blot analysis showed increased MMP-9 when Ca9-22 cells were treated with CSE (B). Ca9-22 Red Hot EVE treatment decreased MMP-9 in the presence or absence of nicotine(B). MMP-13 was increased in cells exposed to CSE or Red Hot EVE with nicotine(C) compared to untreated control cells. Significant differences are shown with p ≤ 0.05.

In smoke-exposed oral squamous cell carcinoma (OSCC), matrix metalloproteinase-9 (MMP-9) facilitates cell invasion by degrading extracellular matrix components like collagen, enabling cancer cells to invade surrounding tissues and metastasize, thus exacerbating disease progression [8]. Western blot analysis evaluated matrix metalloproteinase-9 (MMP-9) expression in Ca9-22 cells. Treatment with 0.05% CSE upregulated MMP-9 expression 2.2-fold compared to controls (P<0.05; Figure 4A). Red Hot EVE with or without nicotine decreased MMP-9 expression by ~1.6-fold (P<0.004) when compared to controls (Figure 4A).

### 3.5 MMP-13 Protein Expression

Western blot analysis assessed MMP-13 expression in Ca9-22 cells. CSE (0.05%) increased MMP-13 expression by 1.7-fold compared to controls (P<0.05). Red Hot EVE with nicotine upregulated MMP-13 by 1.5-fold (P<0.05), while Red Hot EVE without nicotine led to a 1.3-fold decrease (P<0.050in MMP-13 expression. Green Apple EVE, with or without nicotine, had no significant effect on MMP-13 expression (P>0.05) (Figure 4B).

## 4.0 Discussion

Cigarette smoke extract (CSE) significantly increased the invasion of Ca9-22 gingival oral squamous cell carcinoma (OSCC) cells, consistent with cigarette smoking’s role as a major OSCC risk factor, increasing disease risk 7–10 times compared to non-smokers [5,6]. CSE contains over 7,000 chemicals, including polycyclic aromatic hydrocarbons (PAHs) and tobacco-specific nitrosamines (TSNAs), damaging DNA and triggering inflammation [7]. Our results show that 0.05% CSE upregulated the receptor for advanced glycation end-products (RAGE), matrix metalloproteinase-9 (MMP-9), and MMP-13, alongside an increase in nuclear factor kappa B (NF-κB). RAGE, a pattern-recognition receptor, likely activates NF-κB, a key inflammation driver, which boosts MMP production [8]. MMPs degrade the extracellular matrix (ECM), allowing cancer cells to invade and metastasize [29]. This RAGE-NF-κB-MMP pathway matches prior findings in OSCC cells exposed to cigarette smoke, where RAGE inhibition reduced invasion [8]. The robust MMP-9 increase is particularly critical, as MMP-9 degrades collagen, a major ECM component, facilitating metastasis [29]. These findings explain why OSCC has high recurrence rates (up to 50%) and poor survival (20–60%) in smokers [24].

E-cigarette vapor extract (EVE) showed varied effects depending on flavor and nicotine presence, challenging the view of e-cigarettes as a safe alternative to cigarettes. Red Hot EVE, containing cinnamaldehyde, increased Ca9-22 cell invasion with or without nicotine. This suggests that cinnamaldehyde, a spicy flavoring, promotes invasion, potentially through inflammatory pathways, as Red Hot EVE also elevated NF-κB levels. Surprisingly, Red Hot EVE with or without nicotine decreased MMP-9 expression, indicating that invasion may be driven by other MMPs, such as MMP-13, which was upregulated with nicotine. Nicotine likely amplifies these effects via nicotinic acetylcholine receptors (nAChRs), which enhance inflammation and cancer progression [24].

In contrast, Green Apple EVE, likely containing fruit-based flavorings, had no significant effect on invasion, MMP-9, or MMP-13, though it slightly increased NF-κB, suggesting other regulatory enzymes in Ca9-22 cells [14]. E-cigarette aerosols contain volatile organic compounds (VOCs), heavy metals (e.g., nickel), and flavorings, which can inflame or damage oral cells [16]. The flavor-specific responses, with 8.2% adult prevalence and higher among teens, raise concerns about long-term OSCC risk [22].

The distinct effects of CSE and EVE stem from their chemical makeup. CSE’s combustion produces potent carcinogens (PAHs, TSNAs) that strongly activate RAGE and NF-κB, driving invasion [7]. EVE, produced by heating rather than burning, lacks these toxins but includes flavorings and nicotine, which engage different pathways [14]. For example, Red Hot EVE’s cinnamaldehyde may trigger inflammatory responses that promote invasion, amplified by nicotine’s nAChR activation [17,24]. Green Apple EVE’s minimal impact suggests that fruit flavorings are less bioactive in Ca9-22 cells. These differences underscore the need to study e-cigarettes separately from traditional cigarettes, as their mechanisms diverge despite sharing nicotine [15]. The Ca9-22 cell model, derived from gingival OSCC, may be susceptible to RAGE and NF-κB pathways, explaining the robust CSE response compared to EVE’s variable effects [30].

This study’s findings suggest new possible avenues to treat OSCC, especially for patients exposed to cigarettes or e-cigarettes. The RAGE-driven effects of CSE point to RAGE inhibitors, like semi-synthetic glycosaminoglycan ethers (SAGEs), as potential drugs to block invasion in smokers with OSCC [16]. Preclinical studies in other cancers, such as kidney cancer, show RAGE inhibitors reduce tumor spread, supporting their promise [10]. The NF-κB increases with both CSE and Red Hot EVE, suggesting NF-κB inhibitors, like bortezomib, could help reduce inflammation and invasion in OSCC patients [31]. For e-cigarette users, therapies targeting nAChRs or inflammation (e.g., anti-inflammatory drugs) may counteract nicotine’s effects [24]. Since OSCC varies by tumor site (e.g., gingiva vs. tongue), treatments might need tailoring based on whether patients use cigarettes or e-cigarettes [30]. For example, a gingival OSCC patient using Red Hot e-cigarettes could benefit from combined RAGE and NF-κB inhibitors.

With e-cigarette use rising, especially among teens, our findings highlight urgent public health needs. The invasion increases with Red Hot EVE plus nicotine, suggesting that flavored e-cigarettes with nicotine pose OSCC risks, challenging their “safer” label [30]. Regulatory bodies like the U.S. Food and Drug Administration (FDA) have restricted flavored e-cigarettes to curb youth use. Still, our data suggest these rules should also address cancer risks [31,32]. Public health campaigns should educate about e-cigarette risks, emphasizing that flavors like cinnamaldehyde may harm oral health [17]. Clinicians should screen OSCC patients for e-cigarette use, as exposure history could guide treatment [15]. Second-hand e-cigarette aerosol, which also activates RAGE, may pose risks to non-users and needs to be studied [26].

### 4.1 Study Limitations

While Ca9-22 cells are a strong model for gingival OSCC, they may not represent all OSCC types (e.g., tongue or floor-of-mouth tumors). Future studies should test other OSCC cell lines or patient-derived organoids, which mimic the tumor’s natural environment [33]. The 6–24-hour exposures used here reflect acute effects, but OSCC develops over years of tobacco use, so chronic exposure models are needed [15]. Testing only Red Hot and Green Apple EVE limits conclusions about other flavors (e.g., menthol, diacetyl), which may have unique effects [16]. Second-hand smoke and e-cigarette aerosol exposure, linked to RAGE activation, should be explored for OSCC risk in non-users [26]. Finally, in vivo studies (e.g., mouse models) must confirm these findings and test therapies like RAGE or NF-κB inhibitors [10,34].

This study is the first to compare CSE and EVE effects on Ca9-22 OSCC cells, revealing distinct pathways. CSE drives RAGE- and NF-κB-mediated invasion, upregulating MMP-9 and MMP-13, while EVE’s effects depend on flavor and nicotine, with Red Hot EVE promoting invasion via NF-κB and MMP-13. These insights highlight RAGE and NF-κB as therapeutic targets and underscore the need for e-cigarette regulation to reduce OSCC risk. By linking molecular mechanisms to clinical and policy actions, this work advances our understanding of tobacco-related oral cancers.

This study is the first to compare the effects of cigarette smoke extract (CSE) and e-cigarette vapor extract (EVE) on Ca9-22 gingival oral squamous cell carcinoma (OSCC) cells, uncovering unique molecular pathways that drive cancer progression. CSE strongly promotes cell invasion by activating the receptor for advanced glycation end-products (RAGE) and nuclear factor kappa B (NF-κB), which boost matrix metalloproteinases (MMP-2, MMP-9). These MMPs degrade tissue barriers, contributing to OSCC’s high recurrence (up to 50%) and low survival rates (20–60%) in smokers [6,7]. In contrast, EVE’s effects depend on flavor and nicotine. Red Hot EVE (cinnamaldehyde-based) increases invasion with nicotine, driven by NF-κB and MMP-13, but has minimal impact without nicotine. Green Apple EVE has minimal effects, highlighting flavor-specific risks [14,17]. These findings challenge the idea that e-cigarettes are harmless, revealing their potential to exacerbate OSCC in users.

Therapeutic strategies can leverage these insights. For smokers with OSCC, RAGE inhibitors, like semi-synthetic glycosaminoglycan ethers (SAGEs), may block invasion, as seen in other cancers [10]. E-cigarette users, particularly of flavored products with nicotine, could benefit from NF-κB inhibitors, like bortezomib, to reduce inflammation and invasion [33]. Clinicians should screen OSCC patients for cigarette and e-cigarette use to tailor treatments, as exposure influences cancer behavior [15].

Public health efforts are critical, given e-cigarettes’ popularity among teens and adults (8.2% prevalence) [20]. Campaigns should warn about the risks of flavored e-cigarettes, and regulators like the FDA should strengthen flavor bans to protect oral health [32]. Non-users exposed to second-hand e-cigarette aerosol may face risks, as it activates RAGE, though further research is needed [26]. Future studies should validate these findings in animal models and test diverse OSCC cell lines or organoids to capture tumor variability [10,34]. Studies should also explore other e-cigarette flavors (e.g., menthol) and chronic exposures to reflect long-term use [15,16]. Also, future work should include gain/loss of function studies to verify the role of MMP-13 in EVE-induced invasion. Investigating second-hand aerosol effects will clarify non-user risks by revealing how CSE and EVE fuel OSCC. This study paves the way for better treatments and policies to fight tobacco-related oral cancers in a world of changing nicotine products.
